# Evaluation and Management of Hypertensive Disorders of Pregnancy

**DOI:** 10.34067/KID.0000000000000228

**Published:** 2023-10-26

**Authors:** Divya Bajpai, Cristina Popa, Prasoon Verma, Sandi Dumanski, Silvi Shah

**Affiliations:** 1 Department of Nephrology, Seth G.S.M.C & K.E.M. Hospital, Mumbai, India; 2 Department of Internal Medicine - Nephrology, University of Medicine and Pharmacy “Grigore T Popa”, Iasi, Romania; 3 Department of Pediatrics, University of Cincinnati College of Medicine, Cincinnati, Ohio; 4 Division of Neonatology, Cincinnati Children's Hospital Medical Center, Cincinnati, Ohio; 5 Department of Medicine, Cumming School of Medicine, University of Calgary, Calgary, Alberta, Canada; 6 Libin Cardiovascular Institute, Calgary, Alberta, Canada; 7 Alberta Kidney Disease Network, Calgary, Alberta, Canada; 8 Division of Nephrology and Hypertension, University of Cincinnati College of Medicine, Cincinnati, Ohio

**Keywords:** hypertension, pregnancy, preeclampsia, evaluation, treatment

## Abstract

Hypertensive disorders of pregnancy complicate up to 10% of pregnancies and remain the major cause of maternal and neonatal morbidity and mortality. Hypertensive disorders of pregnancy can be classified into four groups depending on the onset of hypertension and the presence of target organ involvement: chronic hypertension, preeclampsia, gestational hypertension, and superimposed preeclampsia on chronic hypertension. Hypertension during pregnancy is associated with a higher risk of cardiovascular disease and kidney failure. Early diagnosis and proper treatment for pregnant women with hypertension remain a priority since this leads to improved maternal and fetal outcomes. Labetalol, nifedipine, methyldopa, and hydralazine are the preferred medications to treat hypertension during pregnancy. In this comprehensive review, we discuss the diagnostic criteria, evaluation, and management of pregnant women with hypertension.

## Introduction

**Figure 1:**
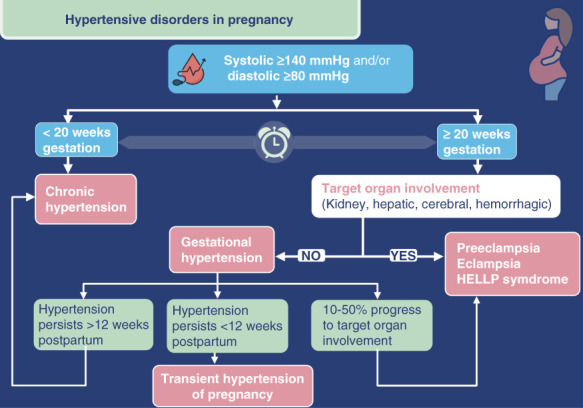
**Spectrum of hypertensive disorders in pregnancy.** HELLP, hemolysis, elevated liver enzymes, low platelets.

Hypertensive disorders of pregnancy affect one in ten pregnant women and are a leading cause of maternal mortality globally, second only to obstetric hemorrhage.^1^ Furthermore, hypertensive disorders of pregnancy also cause significant long-term morbidity for the mother and the offspring.^2^ Therefore, timely diagnosis and optimum management of hypertensive disorders of pregnancy can prove vital in preventing this long-term multimorbidity. The spectrum of hypertensive disorders of pregnancy includes chronic hypertension, gestational hypertension, preeclampsia, and preeclampsia superimposed on chronic hypertension (Figure 1). This narrative review discusses the pathophysiology, definitions, evaluation, and management of hypertensive disorders of pregnancy.

## Pathophysiology of Hypertensive Disorders of Pregnancy

### Hemodynamic Changes in a Healthy Pregnancy and Preeclampsia

To accommodate a growing fetus, maternal hemodynamics undergoes significant changes. The upregulation of the renin–angiotensin–aldosterone system, which begins in the luteal phase, is compounded by hormonal surges after fertilization.^3^ It results in volume expansion *via* salt and water retention. Despite having high levels of renin (up to eight times normal) and aldosterone (up to 20 times normal), there is no rise in BP in normal pregnancy.^4^ This is made possible by pregnancy-related vasodilation and decreased responsiveness of maternal vasculature to vasoconstrictors.^3^ In the early first trimester, estrogen, progesterone, and relaxin surge lead to nitric oxide release, resulting in systemic vasodilation. The vasodilatory action of prostacyclins compounds this effect. Volume expansion and increased ventricular mass cause an increase in the stroke volume. There is physiologic anemia due to volume expansion, and the heart rate rises to compensate for anemia and vasodilatation. Increased stroke volume and heart rate lead to high cardiac output.^5^ Beginning in the early first trimester, the mean arterial pressure drops by approximately 8–10 mm Hg (10% from baseline).^5^ This decline reaches its nadir between the 16th and 20th weeks of gestation, after which it trends toward prepregnancy levels at approximately 40 weeks of gestation. Diastolic pressure shows a more significant decline as compared with systolic pressure. The rise in arterial compliance and venous capacitance in a healthy pregnancy leads to decreased effective plasma volume, resulting in a pregnancy-related decline in BP. Earlier studies suggested that women with preeclampsia have reduced plasma volume.^6^ However, recent evidence is consistent that the suppressed plasma renin activity, higher BP, and subsequent decrease in GFR seen in preeclampsia are consistent with vasoconstriction and overfilled circulation rather than true hypovolemia.^7^

In normal pregnancy, kidneys enlarge, renal blood flow increases by 60%, and the GFR increases by up to 50% in midgestation.^6^ The GFR is approximately 30% lower in women with preeclampsia than in normal pregnancy.^8^ Women with preeclampsia have exaggerated hypercoagulability, dyslipidemia, and insulin resistance compared with normal pregnancy, which puts them at a higher cardiometabolic risk.^9,10^ Women with preexisting vascular diseases, such as hypertension, diabetes, and CKD, are at higher risk of developing preeclampsia. This may be due to preexisting endothelial dysfunction.

### Abnormal Placentation and Preeclampsia

It is now well established that an abnormal placenta is cardinal to the development of preeclampsia. In a healthy pregnancy, the spiral uterine arteries increase in diameter by losing their muscular walls and extending to the myometrium. This process, known as pseudovasculogenesis, transforms them into vessels with large capacitance vessels and low resistance, facilitating high placental blood flow. Failure of this remodeling leads to increased resistance in placental vasculature, causing placental hypoperfusion and ischemia, which forms the basis of preeclampsia.^11,12^

### Angiogenic Imbalance and Maternal Syndrome in Preeclampsia

**Figure 2:**
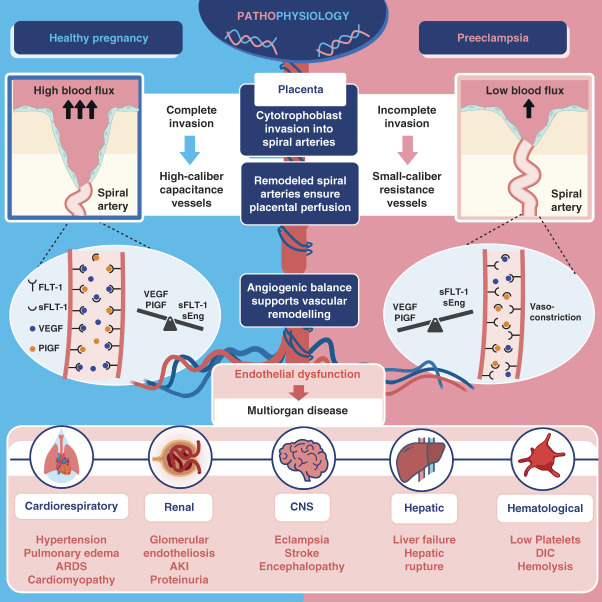
**Pathophysiology of preeclampsia.** PlGF, placental growth factor; sENG, soluble endoglin; sFlt-1, soluble FMS-like tyrosine kinase 1; VEGF, vascular endothelial growth factor; ARDS, acute respiratory distress syndrome; DIC, disseminated intravascular coagulation; CNS, central nervous system.

Placental ischemia and resultant oxidative stress soon cascade to widespread maternal endothelial dysfunction mediated by angiogenic imbalance and inflammatory reaction. Ischemic placenta releases soluble FMS-like tyrosine kinase 1 (sFlt-1) and soluble endoglin, which are antiangiogenic. sFlt-1 binds to vascular endothelial growth factor and placental growth factor (PlGF) and prevents their interaction with the receptors, thus antagonizing their proangiogenic biologic activity. The result is a series of downstream effects culminating in end-organ damage characteristics of preeclampsia (Figure 2).

### Immunologic and Genetic Factors

Maternal immunologic intolerance for fetal antigens might contribute to abnormal placentation in preeclampsia. Lower exposure to paternal antigen (nulliparity, long interpregnancy intervals, and conception after intracytoplasmic injection or oocyte donation) increases the chances of preeclampsia.^13,14^ Women with preeclampsia are found to have a relative deficiency in regulatory T cells that promote immunologic tolerance in healthy pregnancy.^14,15^ In addition, an increased natural killer cell activity is known to recognize HLA class I antigens expressed on the extravillous trophoblast cells.^16^ These alterations, accompanied by increased dendritic cell infiltration in preeclamptic decidual tissue in preeclampsia, led to abnormalities similar to organ rejection.^15,17^ However, there is no definitive evidence of any specific HLA alleles in the pathogenesis of preeclampsia.

Maternal and paternal contributions to fetal genes also play an essential role in preeclampsia by promoting defective placentation. There is a two-fold to five-fold higher risk of preeclampsia in primigravida with a family history of preeclampsia.^18,19^ The risk of preeclampsia is increased in the progeny of a man born from a preeclamptic pregnancy.^20^ As genes for sFlt-1 are located on chromosome 13, a high risk of preeclampsia is seen in women with trisomy 13.^21^ Other potentially significant loci associated with preeclampsia include 12q, 2p13, PAI-1, 2q14.2, 9p13, 2p25, and 2p12.^22,23^

### CKD and Risk of Preeclampsia

The impaired renal reserve is a significant risk factor for preeclampsia. In an experiment by Dupont *et al.*,^24^ uni nephrectomized mice showed impaired adaptation with a lack of increased GFR or plasma volume during early pregnancy, which manifested during late pregnancy as a preeclampsia-like syndrome with hypertension, albuminuria, and glomerular endothelial damage. These mice with single kidneys showed excess placental production of sFlt-1 and failure to upregulate L-kynurenine compared with mice with normal renal reserve. L-kynurenine has a protective role in placental health. A systematic review of 13 studies involving 2862 pregnancies in women with CKD documented an increased risk of hypertensive disorders and maternal mortality (11.5% versus 2% in healthy women) and two times higher risk of adverse fetal events in women with CKD.^25^ Branham *et al.* reported that the rate of preeclampsia and adverse maternal and fetal outcomes increased with the severity of kidney disease. Preeclampsia occurred in 40% of women with mild CKD and 60% of women with moderate to severe CKD.^26^ Increased risk of preeclampsia in kidney donors has been documented to be two-fold to six-fold in various prospective cohorts.^27,28^ Patients with CKD and living kidney donors warrant close monitoring throughout the pregnancy as they are at higher risk for preeclampsia and related adverse events.

## Evaluation of Women with Hypertension in Pregnancy

Evaluating hypertension in pregnancy involves establishing a correct diagnosis of the hypertensive disorders of pregnancy, differentiating between conditions that can closely mimic preeclampsia and timely detection of the associated organ involvement. Diagnostic criteria for various syndromes of hypertensive disorders of pregnancy as per the latest American College of Obstetrics and Gynecology (ACOG) guidelines 2022^29^ are presented in Table 1.

**Table 1:** Diagnostic criteria for hypertensive disorders of pregnancy (adapted from American College of Obstetrics and Gynecology guidelines 2022)

**Preeclampsia**
• Systolic BP of 140 mm Hg or more or diastolic BP of 90 mm Hg or more on two occasions at least 4 h apart after 20 wk of gestation in a woman with previously normal BP
or
• Systolic BP of 160 mm Hg or more or diastolic BP of 110 mm Hg or more. Severe hypertension can be confirmed within a short interval (min) to facilitate timely antihypertensive therapy
and
• Proteinuria: 300 mg or more per 24-h urine collection, protein/creatinine ratio of 0.3 mg/dl or more, or dipstick reading of 2+
**In the absence of proteinuria, new-onset hypertension with the new onset of any of the following (target organ involvement)**
1. Thrombocytopenia: platelet count <100,000×109/L
2. Renal insufficiency: serum creatinine concentrations greater than 1.1 mg/dl or a doubling of the serum creatinine concentration in the absence of other kidney diseases
3. Impaired liver function: elevated blood concentrations of liver transaminases to twice normal concentration
4. Pulmonary edema
5. New-onset headache unresponsive to medication and not accounted for by alternative diagnoses or visual symptoms
**Preeclampsia with severe features** • Systolic BP of 160 mm Hg or more or diastolic BP of 110 mm Hg or more on two occasions at least 4 h apart (unless antihypertensive therapy is initiated before this time) or • Preeclampsia with any of the abovementioned target organ involvement
**Gestational hypertension**
⁃ New onset of systolic BP ≥140 mm Hg and/or diastolic BP ≥90 mm Hg on at least two occasions 4 h apart detected first time after 20 wk of gestation
and
⁃ Absence of proteinuria
⁃ Absence of target organ involvement
**Chronic hypertension**
⁃ Hypertension (systolic BP ≥140 mm Hg and/or diastolic BP ≥90 mm Hg) diagnosed or present before pregnancy or on at least two occasions before 20 wk of gestation. If hypertension is diagnosed *de novo* during pregnancy and persists for more than 12 wk postdelivery; then, it is also considered chronic hypertension
**Chronic hypertension with superimposed preeclampsia**
A patient with chronic hypertension presenting with the following:
• Sudden worsening of BP control and need to escalate antihypertensive therapy
• New onset of proteinuria or a sudden increase in proteinuria in a patient with known proteinuria
• Significant new target organ involvement consistent with preeclampsia ≥20 wk of gestation or postpartum

There are a few close mimics of preeclampsia, which need to be timely differentiated as the management can be significantly different in each^30^ (Table 2). Hemolysis, elevated liver enzymes, low platelet syndrome is often considered a subset of preeclampsia with severe features. However, because few patients with hemolysis, elevated liver enzymes, low platelet syndrome can present without hypertension (10%–12%) and proteinuria (10%–15%), some authors have considered it a separate disorder.^31,32^

**Table 2:** Differentiating features of various disorders that can mimic hypertensive disorders of pregnancy^30^

Clinical Features	Preeclampsia	HELLP Syndrome	HUS	TTP	AFLP	Exacerbation of SLE
Presence of hypertension	100%	85%	80%–90%	20%–75%	50%	If nephritis/antiphospholipid antibody present—80%
Timing during gestation	Diagnosed after 20 wk of gestation, most common to occur in third trimester, 5% can present postpartum	Common in the third trimester	Most commonly occurs near term and worsens postpartum (unlike preeclampsia which improves with delivery)	Onset is earlier than preeclampsia with 65% occurring before the third trimester, hereditary TTP occurs before 20 wk	Most commonly occurs in third trimester, rarely can present up to 4 d postdelivery	Lupus flares can happen in any trimester and in postpartum period
Presenting symptoms	Can be nonspecific as nausea, vomiting, abdominal pain, headache, malaise	Can have abdominal pain and jaundice	Neurologic manifestations are less common as compared with TTP	Can have neurologic manifestations	Can be nonspecific as nausea, vomiting, abdominal pain, headache, malaise. Jaundice is prominent jaundice	Lupus-specific symptoms might be present
**Laboratory parameters**						
Hemolysis	Absent unless complicated by HELLP	Present	Present	Present	Less common	Autoimmune hemolysis may be present
Thrombocytopenia	Present, usually >100,000/mm^3^	Present, usually >20,000/mm^3^	Present, usually >20,000/mm^3^	Present, usually <20,000/mm^3^	Present, usually >50,000/mm^3^	Present, usually >20,000/mm^3^
Kidney dysfunction	Present in severe preeclampsia	Present in 50%	Present in 100%	Present in 30%	Present in 90%–100%	Present in 40%–80%
Hypoglycemia	Absent	Absent	Absent	Absent	Present	Absent
Disseminated intravascular coagulation	Uncommon	Uncommon	Rare	Rare	Common	Rare
Elevated transaminases	Present in severe preeclampsia	Present	Usually mild (<100 IU/L)	Usually mild (<100 IU/L)	Present	Present in liver involvement or antiphospholipid antibody
Elevated bilirubin	Less common	Less common	Indirect hyperbilirubinemia of hemolysis	Indirect hyperbilirubinemia of hemolysis	Present	Less common
Elevated ammonia	Absent	Rare	Absent	Absent	Common	Absent
Serum fibrinogen <300 mg/dl	Rare (only if massive abruption or DIC present)	Rare	Absent	Absent	Common	Absent
ADAMTS13 levels <5%	Absent	Absent	Rare	Present	Absent	Rare
Von Willebrand factor multimers	Absent	Absent	Absent	Increased	Increased	Increased, less common
Abnormal angiogenic markers—high sFlt-1/sENG, low PlGF/VEGF	Present	Present	Absent	Absent	Absent	Absent

HELLP, hemolysis, elevated liver enzymes, low platelet; HUS, hemolytic uremic syndrome; TTP, thrombotic microangiopathic purpura; AFLP, acute fatty liver of pregnancy; SLE, systemic lupus erythematosus; sFlt-1, soluble FMS-like tyrosine kinase 1; sENG, soluble endoglin; PlGF, placental growth factor; VEGF, vascular endothelial growth factor.

### Chronic Hypertension and Superimposed Preeclampsia

Owing to the fall in BP, which reaches a nadir at 16–20 weeks of gestation, a previously hypertensive woman can be misdiagnosed as normotensive during this period if no prepregnancy records are available. Later, when BP returns to normal at term, the rise in BP may be considered preeclampsia. Features that can differentiate preeclampsia are listed in Table 3. However, it is safer that new-onset hypertension is presumed to be due to preeclampsia until proven otherwise, as severe features can rapidly develop, and the patient might progress to eclampsia in a short duration. It is also important to differentiate superimposed preeclampsia from the worsening underlying CKD as the management and complications differ. Classical symptoms (headache, nausea, vomiting, epigastric pain, and visual disturbances) and laboratory abnormalities (hemolysis, thrombocytopenia, liver dysfunction, and high uric acid) are present in preeclampsia. In patients with preeclampsia, proteinuria rapidly increases to a nephrotic range, which might be absent in nonglomerular causes of CKD. Preeclampsia is characterized by improvement up to 12 weeks postpartum, whereas worsening kidney disease might not resolve postdelivery.

**Table 3:** Differentiating features of preeclampsia, chronic hypertension, and superimposed preeclampsia on chronic hypertension

Clinical Features	Preeclampsia	Chronic Hypertension	Superimposed Preeclampsia on Chronic Hypertension
Timing of gestation	New-onset hypertension diagnosed after 20 wk of gestation. (rarely in women with older 40 years/multifetal gestation can occur <20 wk)	Hypertension is diagnosed before 20 wk of gestation, however occasionally masked by early pregnancy BP dip	Sudden increase in previously well-controlled BP after 20 wk of gestation
Proteinuria	Occurs after 20 wk and quick progression to nephrotic range	Can be present before 20 wk (not always present) but usually stable and below 1 g/d	Either new-onset proteinuria or sudden increase to nephrotic range proteinuria in previously stable levels
Symptoms	Classical symptoms of preeclampsia-like visual disturbances, headache, epigastric pain, hyperreflexia may be present	Uncommon	Classical symptoms of preeclampsia-like visual disturbances, headache, epigastric pain, hyperreflexia may be present
Target organ involvement	New-onset organ involvement can be present in severe cases (hemolysis, thrombocytopenia, liver dysfunction, kidney dysfunction, and neurologic involvement)	End-organ damage due to long standing hypertension-like concentric left ventricular hypertrophy, hypertensive retinopathy, kidney disease can be present is stable (serum creatinine may be lower than the baseline over the course of gestation) and does not acutely worsen	New-onset organ involvement can be present in severe cases (hemolysis, thrombocytopenia, liver dysfunction, kidney dysfunction, and neurologic involvement)
Serum uric acid levels	Elevated	Usually normal till kidney function preserved	Elevated
Angiogenic markers	Deranged—high sFlt-1 and sENG, low PlGF, and VEGF	Not deranged	Deranged—high sFlt-1 and sENG, low PlGF, and VEGF
Postpartum course	Typically resolves—BP normalizes >12 wk (rarely can present *de novo* postpartum)	Persists or worsens	Typically resolves; however, worsening of underlying disease can occur

sFlt-1, soluble FMS-like tyrosine kinase 1; sENG, soluble endoglin; PlGF, placental growth factor; VEGF, vascular endothelial growth factor.

### Postpartum Preeclampsia

Rarely, preeclampsia can also present after 48 hours of delivery, known as delayed onset or postpartum preeclampsia.^33^ It is unclear whether this is a subtype of preeclampsia subclinical in the antepartum period or it is a different disease entity. The pattern of angiogenic imbalance (high sFlt-1/PlGF ratio) is similarly seen in women who develop postpartum preeclampsia.^34^ However, prospective studies show differences in the immunologic profile between preeclampsia and postpartum preeclampsia.^35^ Although the immune markers differ, elevated immune markers in the placenta suggest antenatal initiation of pathology in postpartum preeclampsia. Most of these women present with headache; other symptoms can be shortness of breath or impending stroke. Educating women to recognize these symptoms and monitoring BP at least in the first week postpartum is essential.^36^

### Other Medical Disorders Presenting with Hypertension

It is prudent to rule out secondary causes of hypertension while evaluating hypertensive disorders of pregnancy. In women with signs and symptoms of autonomic overactivity (sweating, palpitations, tremors, pallor, and panic attacks), timely work-up for pheochromocytoma is important as it is associated with intrapartum maternal and fetal morbidity and mortality. Tachycardia can also be present in women with hyperthyroidism. Drugs, such as cocaine, amphetamine, and phencyclidine, can cause acute hypertension, and the detailed medication history is warranted for timely diagnosis. Other endocrine causes of hypertension are Cushing syndrome (with signs and symptoms of glucocorticoid excess) and primary hyperaldosteronism (with hypokalemia and metabolic alkalosis). In the presence of symptoms of vascular insufficiency (such as claudications), Takayasu arteritis causing renal artery stenosis must be ruled out with the help of renal artery Doppler. These secondary causes might also lead to accelerated hypertension and superimposed preeclampsia, associated with poor maternal and fetal outcomes.^37^

### Role of Angiogenic Biomarkers in the Evaluation of Hypertension in Pregnancy

Urinary or serum levels of angiogenic biomarkers may help distinguish preeclampsia from other hypertensive disorders in pregnancy. Preeclampsia is associated with increased antiangiogenic factors (sFlt-1 and soluble endoglin) and decreased angiogenic factors (vascular endothelial growth factor and PlGF). These aberrations precede the onset of the clinical signs and correlate with disease severity. Therefore, they can also help in the early detection of preeclampsia and the identification of high-risk patients.^38^ In a prospective cohort study, an sFlt-1:PlGF ratio ≤38 could rule out preeclampsia with a high negative predictive value (99.3%; 95% confidence interval [CI], 97.9% to 99.9%) with good sensitivity (80%) and moderate specificity (78.3%).^39^ National Institute for Health and Care Excellence suggests using PlGF-based tests in association with a clinical assessment to diagnose preeclampsia between 20 and 37 weeks of gestation.^40^ They recommend not to use these tests to decide the timing of delivery. These tests are of greater benefit in high-risk patients and before 37 weeks of gestation. The International Society for the Study of Hypertension in Pregnancy suggests that reduced PlGF <5th percentile for gestational age or increased sFlt-1/PlGF ratio would strengthen the diagnosis of preeclampsia, but they should not be used as a sole criterion in isolation.^41^ The recent Preeclampsia Risk Assessment: Evaluation of Cutoffs to Improve Stratification trial evaluated the utility of sFlt-1:PlGF ratio in 715 women from 18 centers in the United States.^42^ The discriminatory ratio ≥40 yielded 65% positive predictive value and 96% negative predictive value for identifying women with severe preeclampsia within 2 weeks. On the basis of this evidence, Food and Drug Administration cleared this test for risk assessment in preeclampsia.^43^ It is important to emphasize that these tests are not commercially available in most countries and will require standardization globally before widespread clinical use.

### Prevention of Preeclampsia

Lifestyle changes in the preconception period and during gestation have been associated with improved maternal and fetal outcomes.^44^ In a meta-analysis of 44 randomized trials, lifestyle and dietary interventions leading to reduced maternal gestational weight gain reduced the risk of preeclampsia (relative risk, 0.74; 95% CI, 0.60 to 0.92).^45^ In a systematic review of 75 studies evaluating preconception weight loss after bariatric surgery in obese women, the risk of preeclampsia was reduced.^46^ Interpregnancy weight loss also has been shown to reduce the risk of recurrent preeclampsia.^47^ Therefore, all women must be encouraged to maintain a healthy preconception weight, and intragestation weight gain must be optimized, especially in high-risk pregnancies.

Low-dose aspirin reduces platelet activation and maternal inflammation, which mediates preeclampsia by inhibiting platelet thromboxane A2 synthesis while keeping prostacyclin synthesis intact. ACOG and the US preventive task force recommend daily low-dose aspirin (50–150 mg) to all women with a high risk of developing preeclampsia (CKD, chronic hypertension, multiple gestations, diabetes, autoimmune disease, and >1 pregnancy complicated with preeclampsia).^48,49^ It should be started before 16 weeks of gestation on low-dose aspirin.

Epidemiologic data suggest an inverse relationship between calcium intake and hypertension. In a recent meta-analysis of 30 randomized trials, calcium supplementation reduced the risk of preeclampsia (relative risk, 0.49; 95% CI, 0.39 to 0.61) in women with low baseline calcium intake.^50^ On the basis of these results, World Health Organization recommends calcium supplementation (1.5–2 g/d) for women with low calcium intake, particularly those at high risk for preeclampsia.

## Long-Term Outcomes in Women with Hypertension during Pregnancy

**Figure 3:**
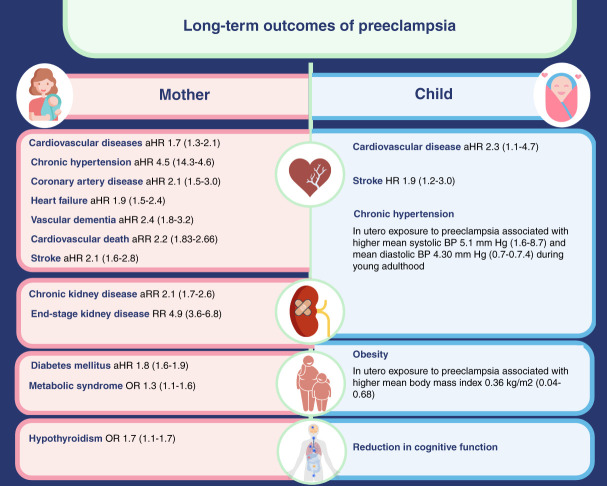
**Long-term outcomes of preeclapmsia.**^74–85^ OR, odds ratio; aHR, adjusted hazard ratio; aRR, adjusted risk ratio; HR, hazard ratio.

It is now proven beyond doubt that preeclampsia is not a pregnancy-limited condition, and it has a significant long-term effect on the well-being of the mother and child (Figure 3). Apart from being at a higher risk of end-organ damage because of preexisting comorbidities (*e.g.*, diabetes, CKD, and obesity), evidence suggests that the endothelial dysfunction that begins during pregnancy persists long after delivery. Studies in mice show preeclampsia-related enrichment of proteins associated with vascular disease, atherosclerosis, and inflammatory response leading to persistent vascular damage.^51^ Thus, along with unmasking the underlying predisposition, preeclampsia also leads to persistent endothelial damage. Numerous prospective studies have documented a higher risk of chronic hypertension, ischemic heart disease,^52^ stroke,^53^ and death from cardiovascular disease^54^ in women with hypertension in pregnancy, even after adjustment for confounding factors. The risk of CKD in the mother is proportional to the severity of hypertension during pregnancy and gets compounded with each preeclamptic pregnancy.^55^ Hence, these women must be counseled for preventive measures, such as weight optimization and regular screening for diabetes, hypertension, and kidney dysfunction. In addition, the history of preeclampsia in the mother must be considered while evaluating the offspring for future cardiovascular risk.

**Figure 4:**
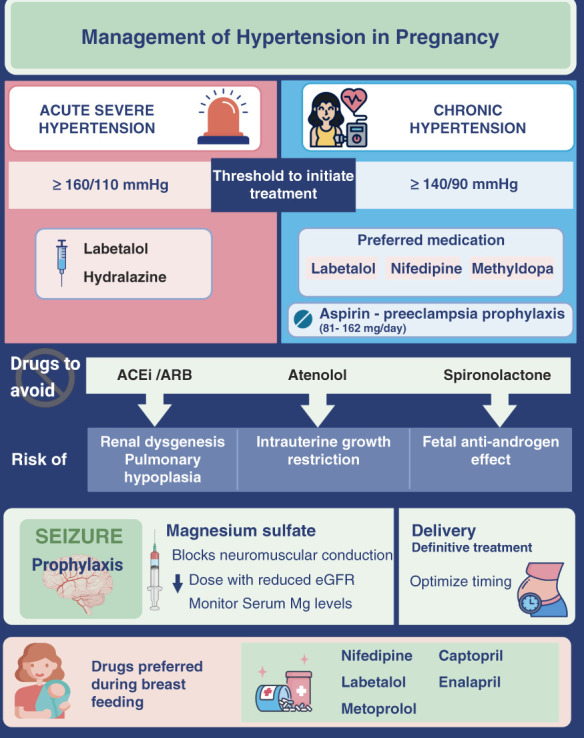
**Treatment of hypertensive disorders of pregnancy.** ACEi, angiotensin-converting enzyme inhibitor; ARB, angiotensin II receptor blocker.

## Management of Hypertension in Pregnancy

Our understanding of the optimum management of hypertensive disorders of pregnancy has evolved over the years. Before initiating treatment for hypertensive disorders of pregnancy, it is vital to consider the risks and benefits for both the mother and the fetus. It can be a tightrope walk as uncontrolled hypertension can cause immediate risk to the mother (stroke, heart failure, and eclampsia). On the other hand, aggressive lowering of BP may compromise fetal perfusion. This risk is especially relevant in preeclampsia, where uteroplacental circulation is compromised. In addition, as all antihypertensive drugs cross the placenta, it is crucial to acknowledge fetal exposure and associated adverse effects.

### Preconception Management of Chronic Hypertension

Preconception considerations for women with chronic hypertension who have optimum BP control on therapy involve evaluating the risk–benefit ratio of switching to a pregnancy-safe drug regimen. It must be a shared decision with the patient, the obstetrician, and the primary care provider. Switching to drugs with an established fetal safety profile preconception is generally preferred as it allays the concerns about teratogenicity and avoids altering the therapy in the first trimester, where hemodynamic changes are increasing.

For women who are on angiotensin-converting enzyme inhibitors and angiotensin II receptor blockers who are contemplating pregnancy and there are no other effective alternatives (proteinuric kidney disease, heart failure, and myocardial infarction), the drugs can be continued till pregnancy detection after discussing risks and benefits with the patients; however, early detection of pregnancy is vital to minimize fetal exposure. The maximum harm occurs in the second and third trimesters as these drugs can interfere with fetal renal hemodynamics. Spironolactone and eplerenone are to be avoided in the preconception period. For women on calcium channel blockers, a switch to extended-release nifedipine is suggested as it has maximum evidence of safety in pregnancy, and those on *β*-blockers can be switched to labetalol, the preferred *β*-blocker in pregnancy. If the woman is on diuretics, they can be continued, but the dose should be kept minimal as it may interfere with the physiologic volume expansion of pregnancy.

### Initiating Antihypertensive Therapy in Pregnant Women

The immediate risk to maternal well-being is the most critical consideration when deciding the timing of initiating antihypertensive treatment in pregnancy.

#### Treatment for Pregnant Women with Severe Hypertension

There is consensus that severe hypertension in the setting of preeclampsia or after 20 weeks of gestation (defined as BP ≥160/110 mm Hg confirmed within 15 minutes) should be treated within 30–60 minutes of diagnosis to reduce the risk of stroke, heart failure, kidney injury, and other severe maternal complications.^29^ In a retrospective report on maternal deaths due to stroke from California, BP above 160 mm Hg systolic was found in 96% of cases and above 110 mm Hg diastolic was found in 65% of cases.^56^ Initial BP reduction should be restricted to 25% over the first 2 hours of therapy as further precipitous lowering can compromise uteroplacental circulation. Close monitoring must continue after achieving a target of 130–150 mm Hg systolic and 80–100 mm Hg diastolic, especially for intrapartum patients (Figure 4).

#### Treatment of Pregnant Women with Mild–Moderate Hypertension

There is less uniformity in the approach to managing mild to moderate hypertension in pregnancy. Guidance from different societies is presented in Table 4. Except for ACOG, most other organizations endorse a more aggressive antihypertensive approach in pregnancy with targets similar to the general population. More aggressive treatment has consistently been shown to prevent the development of severe hypertension, as documented by a Cochrane review of 63 trials^57^ and by the Control of Hypertension in Pregnancy Study (CHIPS), in which the mean BP in the tight control group was 133/85 mm Hg.^58^ However, the CHIPS primary outcome (risk of perinatal loss or high-level neonatal care for >48 hours) and secondary outcome (serious maternal complications) were not different in the tight control group. In a *post hoc* analysis of CHIPS, even after adjustment for preeclampsia, patients with severe hypertension were at a higher risk of perinatal loss or high-level neonatal care for >48 hours, preterm birth, birth weight <10th percentile, and elevated liver enzymes with symptoms.^59^ In CHIPS, delaying initiation of antihypertensive therapy until 24 weeks of gestation was associated with improved birth weights. However, this was counterbalanced by an increased iatrogenic preterm birth due to severe hypertension. Thus, there was no overall effect on perinatal death or morbidity.^58^

**Table 4:** BP thresholds for the management of hypertension in pregnancy

Guideline	BP Threshold for Initiating Treatment, mm Hg	Treatment Target, mm Hg	Remarks
ACOG 2022^68^	≥140/90 mm Hg for chronic hypertension ≥160/110 mm Hg for acute hypertension	Not specified	Recommends continuation of preexisting antihypertensive therapy after informed discussion with the patient
International Society for the Study of Hypertension in Pregnancy 2018^69^	For chronic hypertension— ≥140/90 mm Hg in office ≥135/85 mm Hg at home For acute hypertension— ≥140/90 mm Hg	110–140 mm Hg/85 mm Hg	—
National Institute for Health and Care Excellence 2019^70^	≥140/90 mm Hg	≤135/85 mm Hg	Continue preexisting therapy unless <110/70 mm Hg or symptomatic hypotension
Society of Obstetricians and Gynecologists, Canada, 2020^71^	≥140/90 mm Hg	Diastolic BP <85 mm Hg For chronic hypertension <140/90 mm Hg if comorbidities present	—
European Society of Cardiology 2018^72^	≥140/90 mm Hg for acute hypertension For chronic hypertension— ≥150/95 mm Hg; ≥140/90 mm Hg with end-organ damage	Not specified	—

ACOG, American College of Obstetrics and Gynecology.

The benefit of lowering BP <140/90 mm Hg was shown in pregnant women with chronic hypertension in the recent Control of Mild Hypertension During Pregnancy Trial.^60^ Active treatment reduced the composite outcome of preeclampsia with severe features, medically indicated preterm birth <35 weeks, abruption, or fetal or neonatal death. After this, ACOG has amended its recommendations to initiate antihypertensives at BP 140/90 mm Hg in pregnant women with chronic hypertension. The threshold for preeclampsia is still unclear, but there is an increasing trend toward aggressive treatment. This is especially important for resource-limited settings where the capacity to deal with rapidly developing complications might be limited. According to their position statement (2022), the American Heart Association endorses treatment of nonsevere hypertension during pregnancy to targets similar to those recommended in nonpregnant individuals after informed decision making with the patients.^1^ Home monitoring of BP is encouraged, and frequent titration may be needed due to hemodynamic physiologic changes and hyperemesis that occur during pregnancy.

### Drugs Used for the Treatment of Hypertension in Pregnancy

#### Acute Treatment of Severe Hypertension

Urgent treatment with intravenous (IV) drugs is recommended in women with severe hypertension or signs of impending eclampsia (headache, visual blurring, and epigastric pain). Labetalol is the preferred first-line drug because of its rapid onset of action (<5 minutes), good efficacy, and side effect profile (Table 5). Relative contraindications to *β*-blocker use are asthma and maternal bradycardia. Women with concomitant heart disease require continuous cardiac monitoring. IV hydralazine can also be used if there is no response or intolerance to labetalol. In selected cases where IV access is delayed, nifedipine immediate release 10 mg can be given with the caution of unpredictable and precipitous response (less with extended-release preparations). The maternal and fetal heart rates must be closely monitored throughout the treatment of severe hypertension.

**Table 5:** Pharmacotherapy of hypertension in pregnancy

Drug	Dosage
**Acute therapy for severe hypertension**	
Labetalol (combined *α* and *β*-blocker)	Bolus regimen: initial dose: 20 mg IV gradually over 2 min Repeat BP >10 min, give 40 mg IV over 2 min To escalate dose with every 10 min BP measurements. If BP target is not achieved with a cumulative maximum dose of 300 mg, switch to another drug Infusion regimen: IV infusion 1–2 mg/min after 20 mg bolus dose. Adjust the dose every 15 min to achieve a maximum cumulative dose of 300 mg
Hydralazine (direct arteriolar vasodilator)	Initial dose: 5 mg IV gradually over 1–2 min Repeat BP at 20 min: give 5 or 10 mg IV over 2 min If BP uncontrolled at 40 min, give 5–10 mg IV over 2 min. Cumulative maximum dose is 20–30 mg per treatment event
Nifedipine immediate release (calcium channel blocker)	10 mg oral. Can be repeated at 20-min intervals Use must be limited to only patients with hypertensive emergency and no IV access. It may cause a precipitous fall in BP and/or fetal heart rate deceleration
Nifedipine extended release	30 mg oral. Can be repeated at 60-min intervals Slower onset of action
**Oral maintenance therapy**	
Labetalol	Initial dose: 100 mg BD. Increase by 100 mg BD over 2–3 d. Max dose 2400 mg Caution: rare cases of maternal hepatotoxicity are reported^73^
Nifedipine extended release	Initial dose: 30–60 mg OD. Increase by 30 mg over 7–14 d. Higher doses can be better given in divided doses max dose 120 mg
Methyldopa (centrally acting *α*-agonist)	Initial dose: 250 mg 2–3 times/d. Increase by 250 mg over 2 d. Max dose 3000 mg Adverse effects: sedation, postpartum depression
Hydralazine	Initial dose: 10 mg four times/d. Increase by 10–25 mg over 2–5 d. Max dose 200 mg Adverse effects: reflex tachycardia. Better to combine with *β*-blockers
**Drugs to be avoided in pregnancy**	
Atenolol (*β* one selective *β*-blocker)	Associated with decreased fetal and placental weight and intrauterine growth secretion, extensively secreted in breast milk, and *β* blockade in nursing infants is reported
Propranolol (nonselective *β* blocker)	Antagonism of *β*-2 receptors, which can cause uterine irritability, has been associated with decreased fetal and placental weight
Renin-angiotensin inhibitors (ACEi, ARBs, and direct renin inhibitors)	Associated with fetal renal abnormalities, especially when exposure occurs in the latter half of pregnancy. Reports of cardiovascular and central nervous system malformations are present
Mineralocorticoid receptor antagonists (spironolactone, eplerenone)	Competitive inhibition of aldosterone binding to the mineralocorticoid receptor leads to increased epithelial sodium channel degradation causing reduced sodium reabsorption and potassium excretion. It can cause volume depletion and hypotension Spironolactone also binds to androgen and progesterone receptors, and feminization of male fetus is a concern
Nitroprusside (vasodilator)	Associated with fetal cyanide poisoning It can be used as a last resort in hypertensive emergency; however, dose and duration must be restricted to minimum

IV, intravenous; ACEi, angiotensin-converting enzyme inhibitor; ARB, angiotensin II receptor blocker.

#### Maintenance Therapy with Oral Drugs

All antihypertensive drugs cross the placenta, and evidence for safety and efficacy is limited. According to the Cochrane review, no evidence suggests that one drug is superior to others.^57^ *β*-blockers are the preferred first-line drugs, with labetalol being the drug of choice as it preserves better uteroplacental circulation (fewer data for the use of carvedilol and metoprolol). However, atenolol is contraindicated due to its association with intrauterine growth restriction. *α*-methyldopa has stood the test of time with documented long-term safety profile. However, it has less potency, slower onset of action (3–6 hours), and sedative side effects. As the availability of methyldopa may be restricted, clonidine is another adrenergic agonist which can be used. However, it can cause rebound hypertension which needs monitoring. Nifedipine and nicardipine are commonly used calcium channel blockers in pregnancy. Sustained-release formulations are preferred due to the predictable action profile. Nondihydropyridine calcium antagonists (verapamil and diltiazem) should be avoided with *β*-blockers for the risk of bradycardia and arteriovenous conduction block. Hydralazine can be combined with a *β*-blocker in a resistant patient as it causes reflex tachycardia. Fluid retention is one dose-limiting side effect. Diuretics must be restricted to patients with heart failure and pulmonary congestion as they cause volume depletion and can compromise fetal circulation. However, they can be used in women with salt-sensitive chronic hypertension at lower dosages.^61^ Drugs that are better avoided during pregnancy are listed in Table 5.

### Diet and Physical Activity

Women are advised to consume a healthy diet without significant salt restriction. Salt restriction can lead to intravascular volume constriction. Evidence to support the Dietary Approaches to Stop Hypertension diet during pregnancy is scarce.^62^ There is no evidence to support restricting physical activity in stable women with mild–moderate hypertension. However, patients with severe features near term may benefit from rest as it may improve uteroplacental circulation and prevent exacerbation of hypertension. This advice must be individualized based on the patient's profile and access to medical care.

### Other Considerations in the Management of Preeclampsia

#### Timing of Delivery

Delivery of the placenta is the only definitive treatment for preeclampsia, which prevents disease progression and its associated complications. The delivery timing needs to be individualized based on the gestational age, BP levels, and maternal and fetal conditions. Preeclampsia with severe features is an indication of immediate delivery regardless of gestational age because of the high risk of maternal morbidity. In patients with nonsevere features, delivery can be planned at 37 weeks with careful monitoring of the maternal and fetal conditions. Maternal monitoring includes BP measurements at least twice a day and laboratory parameters (complete hemogram and renal and liver function) at least twice a week. Fetal monitoring includes daily fetal movement counts, twice weekly nonstress tests, and a biophysical profile. Antenatal betamethasone must be given to women with preeclampsia at <34 weeks of gestation.

#### Seizure Prophylaxis

Magnesium sulfate is shown in randomized studies to be effective in the prevention and treatment of eclampsia in women with preeclampsia with severe features.^63^ In meta-analyses of randomized trials, magnesium sulfate was safer and more effective than phenytoin,^64^ diazepam,^65^ or lytic cocktail^66^ for eclamptic women. Magnesium sulfate does not prevent the progression of other organ involvement because of preeclampsia unrelated to seizures. The role of seizure prophylaxis in preeclampsia without severe features is unclear, and ACOG recommends individualizing the decision on the basis of the risk–benefit ratio.^29^ As magnesium is renally excreted, a reduced maintenance dose is recommended for women with reduced GFR. Careful monitoring for clinical signs of magnesium toxicity and magnesium levels is warranted.

### Postpartum Management

BP tends to reach the preconception level by 6–12 weeks postpartum. However, up to 20% of women can have postpartum hypertension (including new-onset preeclampsia) within 6 weeks of delivery.^34^ Transient hypertension can be related to volume changes or effects of drugs (non steroidal anti-inflamatory drugs and ergot derivatives). All patients require detailed evaluation and close monitoring for target organ involvement (headache and visual blurring). Target BP can be similar to the general population, but the BP goal needs to be individualized. Dosages of antihypertensive drugs need to be adjusted and subsequently stopped in most patients. Secretion of antihypertensive medications during lactation is an important consideration. Nifedipine, labetalol, lisinopril, and metoprolol are safe during lactation.

It is important to remember that hypertension in pregnancy offers a valuable opportunity to diagnose preexisting CKD, which can be as high as 19%.^67^ As noted above, preeclampsia is associated with an increased risk of ongoing kidney injury. Thus, careful monitoring of kidney function is necessary for all women in the postpartum period.

In conclusion, this review presents the current best practices in evaluating and managing pregnant women with hypertension. The management of hypertensive disorders of pregnancy requires multidisciplinary management. Women with hypertensive complications in pregnancy must be counseled postnatally regarding the increased risk of cardiovascular complications and kidney disease. It is critical for timely diagnosis and optimal management of hypertension during pregnancy because of improved maternal and fetal outcomes.
